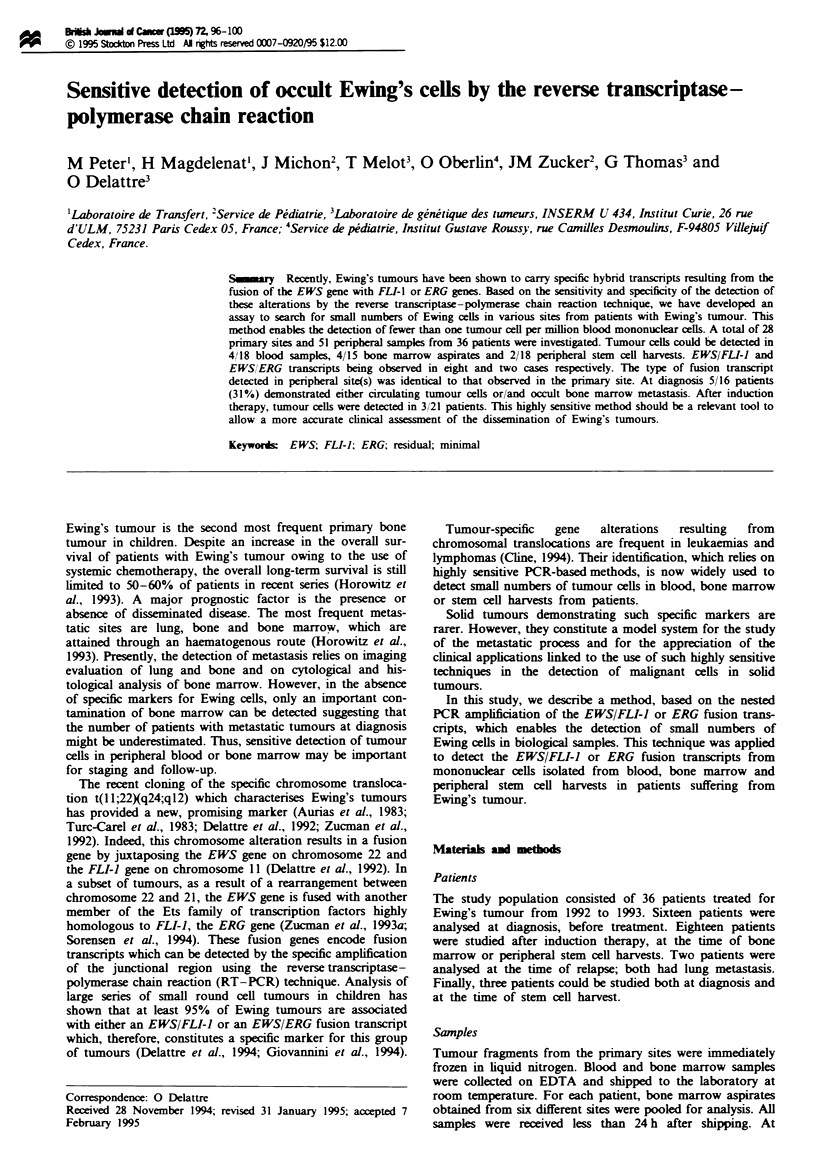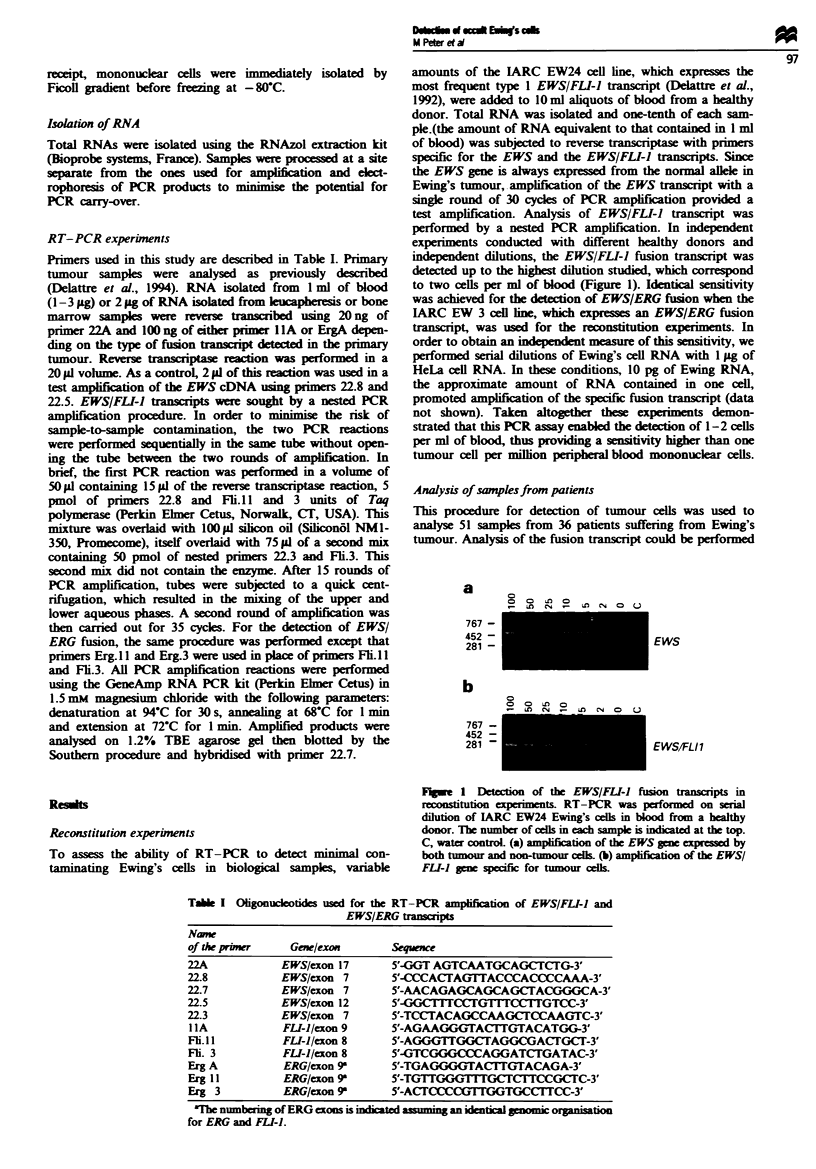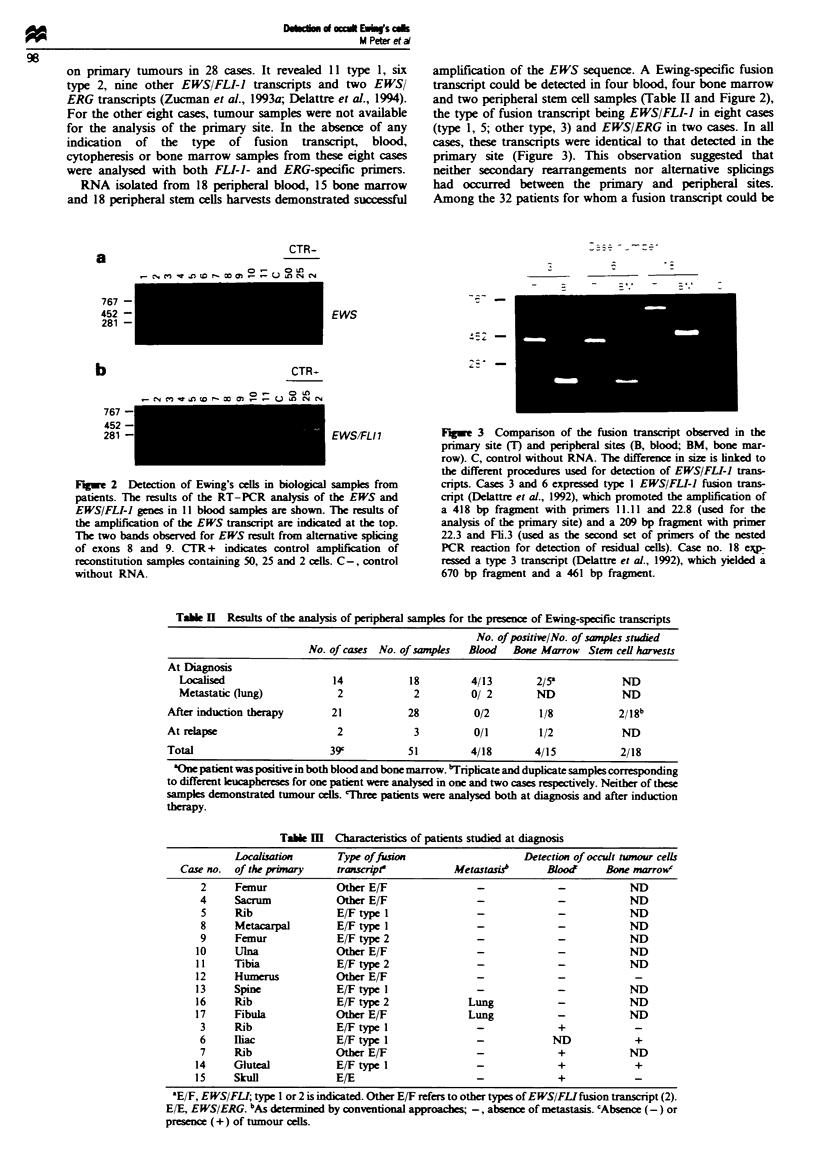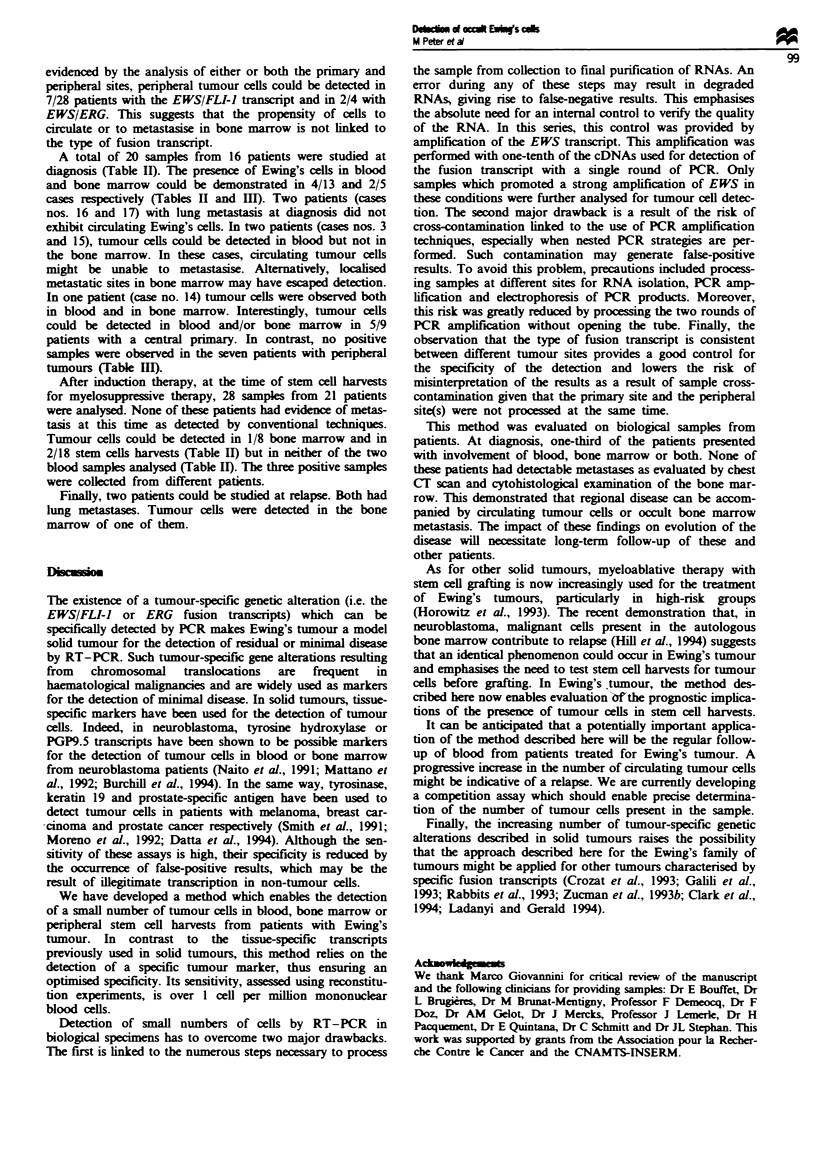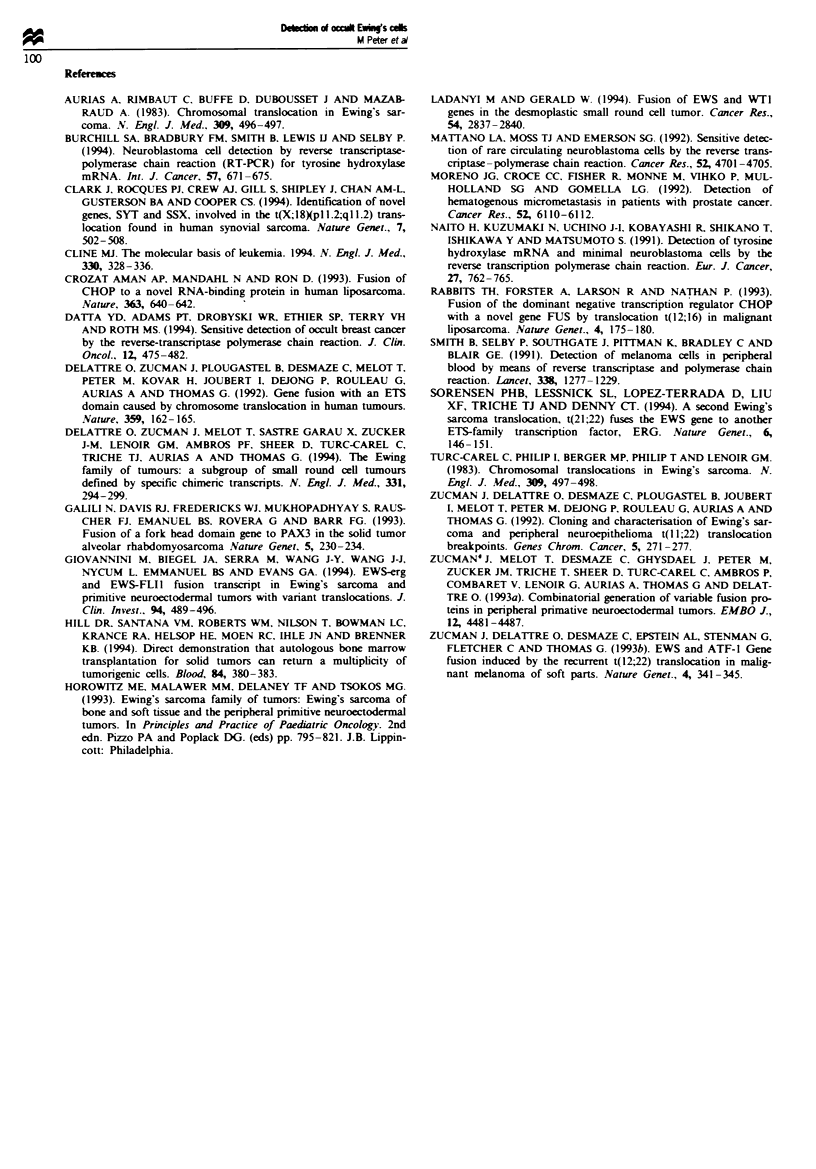# Sensitive detection of occult Ewing's cells by the reverse transcriptase-polymerase chain reaction.

**DOI:** 10.1038/bjc.1995.283

**Published:** 1995-07

**Authors:** M. Peter, H. Magdelenat, J. Michon, T. Melot, O. Oberlin, J. M. Zucker, G. Thomas, O. Delattre

**Affiliations:** Laboratoire de Transfert, INSERM U 434, Institut Curie, Paris, France.

## Abstract

**Images:**


© 1995 Stockton Press Ltd All rights reserved 0007-0920/95 $12.00

### Sensitive detection of occult Ewing's cells by the reverse transcriptase-polymerase chain reaction

M Peter[1], H Magdelenat[1], J Michon[2], T Melot[3], O Oberlin[4], JM Zucker[2], G Thomas[3] and O Delattre[3]

[1]*Laboratoire de Transfert,* [2]*Service de Pédiatrie,* [3]*Laboratoire de génétique des tumeurs, INSERM U 434, Institut Curie, 26 rue d'ULM, 75231 Paris Cedex 05, France;* [4]*Service de pédiatrie, Institut Gustave Roussy, rue Camilles Desmoulins, F-94805 Villejuif Cedex, France.*

**Summary** Recently, Ewing's tumours have been shown to carry specific hybrid transcripts resulting from the fusion of the *EWS* gene with *FLI*-1 or *ERG* genes. Based on the sensitivity and specificity of the detection of these alterations by the reverse transcriptase-polymerase chain reaction technique, we have developed an assay to search for small numbers of Ewing cells in various sites from patients with Ewing's tumour. This method enables the detection of fewer than one tumour cell per million blood mononuclear cells. A total of 28 primary sites and 51 peripheral samples from 36 patients were investigated. Tumour cells could be detected in 4/18 blood samples, 4/15 bone marrow aspirates and 2/18 peripheral stem cell harvests. *EWS/FLI-1* and *EWS/ERG* transcripts being observed in eight and two cases respectively. The type of fusion transcript detected in peripheral site(s) was identical to that observed in the primary site. At diagnosis 5/16 patients (31%) demonstrated either circulating tumour cells or/and occult bone marrow metastasis. After induction therapy, tumour cells were detected in 3/21 patients. This highly sensitive method should be a relevant tool to allow a more accurate clinical assessment of the dissemination of Ewing's tumours.

**Keywords:** *EWS*; *FLI-1*; *ERG*; residual; minimal

Ewing's tumour is the second most frequent primary bone tumour in children. Despite an increase in the overall survival of patients with Ewing's tumour owing to the use of systemic chemotherapy, the overall long-term survival is still limited to 50-60% of patients in recent series (Horowitz *et al.*, 1993). A major prognostic factor is the presence or absence of disseminated disease. The most frequent metastatic sites are lung, bone and bone marrow, which are attained through an haematogenous route (Horowitz *et al.*, 1993). Presently, the detection of metastasis relies on imaging evaluation of lung and bone and on cytological and histological analysis of bone marrow. However, in the absence of specific markers for Ewing cells, only an important contamination of bone marrow can be detected suggesting that the number of patients with metastatic tumours at diagnosis might be underestimated. Thus, sensitive detection of tumour cells in peripheral blood or bone marrow may be important for staging and follow-up.

The recent cloning of the specific chromosome translocation t(11;22)(q24;q12) which characterises Ewing's tumours has provided a new, promising marker (Aurias *et al.*, 1983; Turc-Carel *et al.*, 1983; Delattre *et al.*, 1992; Zucman *et al.*, 1992). Indeed, this chromosome alteration results in a fusion gene by juxtaposing the *EWS* gene on chromosome 22 and the *FLI-1* gene on chromosome 11 (Delattre *et al.*, 1992). In a subset of tumours, as a result of a rearrangement between chromosome 22 and 21, the *EWS* gene is fused with another member of the Ets family of transcription factors highly homologous to *FLI-1*, the *ERG* gene (Zucman *et al.*, 1993*a*; Sorensen *et al.*, 1994). These fusion genes encode fusion transcripts which can be detected by the specific amplification of the junctional region using the reverse transcriptase-polymerase chain reaction (RT-PCR) technique. Analysis of large series of small round cell tumours in children has shown that at least 95% of Ewing tumours are associated with either an *EWS/FLI-1* or an *EWS/ERG* fusion transcript which, therefore, constitutes a specific marker for this group of tumours (Delattre *et al.*, 1994; Giovannini *et al.*, 1994).

Correspondence: O Delattre
Received 28 November 1994; revised 31 January 1995; accepted 7 February 1995

Tumour-specific gene alterations resulting from chromosomal translocations are frequent in leukaemias and lymphomas (Cline, 1994). Their identification, which relies on highly sensitive PCR-based methods, is now widely used to detect small numbers of tumour cells in blood, bone marrow or stem cell harvests from patients.

Solid tumours demonstrating such specific markers are rarer. However, they constitute a model system for the study of the metastatic process and for the appreciation of the clinical applications linked to the use of such highly sensitive techniques in the detection of malignant cells in solid tumours.

In this study, we describe a method, based on the nested PCR amplificiation of the *EWS/FLI-1* or *ERG* fusion transcripts, which enables the detection of small numbers of Ewing cells in biological samples. This technique was applied to detect the *EWS/FLI-1* or *ERG* fusion transcripts from mononuclear cells isolated from blood, bone marrow and peripheral stem cell harvests in patients suffering from Ewing's tumour.

### Materials and methods

### Patients

The study population consisted of 36 patients treated for Ewing's tumour from 1992 to 1993. Sixteen patients were analysed at diagnosis, before treatment. Eighteen patients were studied after induction therapy, at the time of bone marrow or peripheral stem cell harvests. Two patients were analysed at the time of relapse; both had lung metastasis. Finally, three patients could be studied both at diagnosis and at the time of stem cell harvest.

### Samples

Tumour fragments from the primary sites were immediately frozen in liquid nitrogen. Blood and bone marrow samples were collected on EDTA and shipped to the laboratory at room temperature. For each patient, bone marrow aspirates obtained from six different sites were pooled for analysis. All samples were received less than 24 h after shipping. At receipt, mononuclear cells were immediately isolated by Ficoll gradient before freezing at $-80°C$.

### *Isolation of RNA*

Total RNAs were isolated using the RNAzol extraction kit (Bioprobe systems, France). Samples were processed at a site separate from the ones used for amplification and electrophoresis of PCR products to minimise the potential for PCR carry-over.

### *RT-PCR experiments*

Primers used in this study are described in Table I. Primary tumour samples were analysed as previously described (Delattre *et al.*, 1994). RNA isolated from 1 ml of blood (1-3 μg) or 2 μg of RNA isolated from leucapheresis or bone marrow samples were reverse transcribed using 20 ng of primer 22A and 100 ng of either primer 11A or ErgA depending on the type of fusion transcript detected in the primary tumour. Reverse transcriptase reaction was performed in a 20 μl volume. As a control, 2 μl of this reaction was used in a test amplification of the *EWS* cDNA using primers 22.8 and 22.5. *EWS/FLI-1* transcripts were sought by a nested PCR amplification procedure. In order to minimise the risk of sample-to-sample contamination, the two PCR reactions were performed sequentially in the same tube without opening the tube between the two rounds of amplification. In brief, the first PCR reaction was performed in a volume of 50 μl containing 15 μl of the reverse transcriptase reaction, 5 pmol of primers 22.8 and Fli.11 and 3 units of *Taq* polymerase (Perkin Elmer Cetus, Norwalk, CT, USA). This mixture was overlaid with 100 μl silicon oil (Siliconöl NM1-350, Promecome), itself overlaid with 75 μl of a second mix containing 50 pmol of nested primers 22.3 and Fli.3. This second mix did not contain the enzyme. After 15 rounds of PCR amplification, tubes were subjected to a quick centrifugation, which resulted in the mixing of the upper and lower aqueous phases. A second round of amplification was then carried out for 35 cycles. For the detection of *EWS/ERG* fusion, the same procedure was performed except that primers Erg.11 and Erg.3 were used in place of primers Fli.11 and Fli.3. All PCR amplification reactions were performed using the GeneAmp RNA PCR kit (Perkin Elmer Cetus) in 1.5 mM magnesium chloride with the following parameters: denaturation at 94°C for 30 s, annealing at 68°C for 1 min and extension at 72°C for 1 min. Amplified products were analysed on 1.2% TBE agarose gel then blotted by the Southern procedure and hybridised with primer 22.7.

### Results

### *Reconstitution experiments*

To assess the ability of RT-PCR to detect minimal contaminating Ewing's cells in biological samples, variable amounts of the IARC EW24 cell line, which expresses the most frequent type 1 *EWS/FLI-1* transcript (Delattre *et al.*, 1992), were added to 10 ml aliquots of blood from a healthy donor. Total RNA was isolated and one-tenth of each sample.(the amount of RNA equivalent to that contained in 1 ml of blood) was subjected to reverse transcriptase with primers specific for the *EWS* and the *EWS/FLI-1* transcripts. Since the *EWS* gene is always expressed from the normal allele in Ewing's tumour, amplification of the *EWS* transcript with a single round of 30 cycles of PCR amplification provided a test amplification. Analysis of *EWS/FLI-1* transcript was performed by a nested PCR amplification. In independent experiments conducted with different healthy donors and independent dilutions, the *EWS/FLI-1* fusion transcript was detected up to the highest dilution studied, which correspond to two cells per ml of blood (Figure 1). Identical sensitivity was achieved for the detection of *EWS/ERG* fusion when the IARC EW 3 cell line, which expresses an *EWS/ERG* fusion transcript, was used for the reconstitution experiments. In order to obtain an independent measure of this sensitivity, we performed serial dilutions of Ewing's cell RNA with 1 μg of HeLa cell RNA. In these conditions, 10 pg of Ewing RNA, the approximate amount of RNA contained in one cell, promoted amplification of the specific fusion transcript (data not shown). Taken altogether these experiments demonstrated that this PCR assay enabled the detection of 1-2 cells per ml of blood, thus providing a sensitivity higher than one tumour cell per million peripheral blood mononuclear cells.

### *Analysis of samples from patients*

This procedure for detection of tumour cells was used to analyse 51 samples from 36 patients suffering from Ewing's tumour. Analysis of the fusion transcript could be performed

**Figure 1** Detection of the *EWS/FLI-1* fusion transcripts in reconstitution experiments. RT-PCR was performed on serial dilution of IARC EW24 Ewing's cells in blood from a healthy donor. The number of cells in each sample is indicated at the top. C, water control. (**a**) amplification of the *EWS* gene expressed by both tumour and non-tumour cells. (**b**) amplification of the *EWS/FLI-1* gene specific for tumour cells.

**Table I** Oligonucleotides used for the RT-PCR amplification of *EWS/FLI-1* and *EWS/ERG* transcripts

| *Name of the primer* | *Gene/exon* | *Sequence* |
|---|---|---|
| 22A | *EWS*/exon 17 | 5'-GGT AGTCAATGCAGCTCTG-3' |
| 22.8 | *EWS*/exon 7 | 5'-CCCACTAGTTACCCACCCCAAA-3' |
| 22.7 | *EWS*/exon 7 | 5'-AACAGAGCAGCAGCTACGGGCA-3' |
| 22.5 | *EWS*/exon 12 | 5'-GGCTTTCCTGTTTCCTTGTCC-3' |
| 22.3 | *EWS*/exon 7 | 5'-TCCTACAGCCAAGCTCCAAGTC-3' |
| 11A | *FLI-1*/exon 9 | 5'-AGAAGGGTACTTGTACATGG-3' |
| Fli.11 | *FLI-1*/exon 8 | 5'-AGGGTTGGCTAGGCGACTGCT-3' |
| Fli. 3 | *FLI-1*/exon 8 | 5'-GTCGGGCCCAGGATCTGATAC-3' |
| Erg A | *ERG*/exon 9* | 5'-TGAGGGGTACTTGTACAGA-3' |
| Erg 11 | *ERG*/exon 9* | 5'-TGTTGGGTTTGCTCTTCCGCTC-3' |
| Erg 3 | *ERG*/exon 9* | 5'-ACTCCCCGTTGGTGCCTTCC-3' |

*The numbering of ERG exons is indicated assuming an identical genomic organisation for *ERG* and *FLI-1*.

on primary tumours in 28 cases. It revealed 11 type 1, six type 2, nine other *EWS/FLI-1* transcripts and two *EWS/ERG* transcripts (Zucman *et al.*, 1993*a*; Delattre *et al.*, 1994). For the other eight cases, tumour samples were not available for the analysis of the primary site. In the absence of any indication of the type of fusion transcript, blood, cytopheresis or bone marrow samples from these eight cases were analysed with both *FLI-1*- and *ERG*-specific primers.

RNA isolated from 18 peripheral blood, 15 bone marrow and 18 peripheral stem cells harvests demonstrated successful amplification of the *EWS* sequence. A Ewing-specific fusion transcript could be detected in four blood, four bone marrow and two peripheral stem cell samples (Table II and Figure 2), the type of fusion transcript being *EWS/FLI-1* in eight cases (type 1, 5; other type, 3) and *EWS/ERG* in two cases. In all cases, these transcripts were identical to that detected in the primary site (Figure 3). This observation suggested that neither secondary rearrangements nor alternative splicings had occurred between the primary and peripheral sites. Among the 32 patients for whom a fusion transcript could be

**Figure 2** Detection of Ewing's cells in biological samples from patients. The results of the RT–PCR analysis of the *EWS* and *EWS/FLI-1* genes in 11 blood samples are shown. The results of the amplification of the *EWS* transcript are indicated at the top. The two bands observed for *EWS* result from alternative splicing of exons 8 and 9. CTR+ indicates control amplification of reconstitution samples containing 50, 25 and 2 cells. C–, control without RNA.

**Figure 3** Comparison of the fusion transcript observed in the primary site (T) and peripheral sites (B, blood; BM, bone marrow). C, control without RNA. The difference in size is linked to the different procedures used for detection of *EWS/FLI-1* transcripts. Cases 3 and 6 expressed type 1 *EWS/FLI-1* fusion transcript (Delattre *et al.*, 1992), which promoted the amplification of a 418 bp fragment with primers 11.11 and 22.8 (used for the analysis of the primary site) and a 209 bp fragment with primer 22.3 and Fli.3 (used as the second set of primers of the nested PCR reaction for detection of residual cells). Case no. 18 expressed a type 3 transcript (Delattre *et al.*, 1992), which yielded a 670 bp fragment and a 461 bp fragment.

**Table II** Results of the analysis of peripheral samples for the presence of Ewing-specific transcripts

| | *No. of cases* | *No. of samples* | *No. of positive/No. of samples studied* Blood | Bone Marrow | Stem cell harvests |
|---|---|---|---|---|---|
| At Diagnosis | | | | | |
| Localised | 14 | 18 | 4/13 | 2/5[a] | ND |
| Metastatic (lung) | 2 | 2 | 0/ 2 | ND | ND |
| After induction therapy | 21 | 28 | 0/2 | 1/8 | 2/18[b] |
| At relapse | 2 | 3 | 0/1 | 1/2 | ND |
| Total | 39[c] | 51 | 4/18 | 4/15 | 2/18 |

[a]One patient was positive in both blood and bone marrow. [b]Triplicate and duplicate samples corresponding to different leucaphereses for one patient were analysed in one and two cases respectively. Neither of these samples demonstrated tumour cells. [c]Three patients were analysed both at diagnosis and after induction therapy.

**Table III** Characteristics of patients studied at diagnosis

| *Case no.* | *Localisation of the primary* | *Type of fusion transcript*[a] | *Metastasis*[b] | *Detection of occult tumour cells* *Blood*[c] | *Bone marrow*[c] |
|---|---|---|---|---|---|
| 2 | Femur | Other E/F | – | – | ND |
| 4 | Sacrum | Other E/F | – | – | ND |
| 5 | Rib | E/F type 1 | – | – | ND |
| 8 | Metacarpal | E/F type 1 | – | – | ND |
| 9 | Femur | E/F type 2 | – | – | ND |
| 10 | Ulna | Other E/F | – | – | ND |
| 11 | Tibia | E/F type 2 | – | – | ND |
| 12 | Humerus | Other E/F | – | – | – |
| 13 | Spine | E/F type 1 | – | – | ND |
| 16 | Rib | E/F type 2 | Lung | – | ND |
| 17 | Fibula | Other E/F | Lung | – | ND |
| 3 | Rib | E/F type 1 | – | + | – |
| 6 | Iliac | E/F type 1 | – | ND | + |
| 7 | Rib | Other E/F | – | + | ND |
| 14 | Gluteal | E/F type 1 | – | + | + |
| 15 | Skull | E/E | – | + | – |

[a]E/F, *EWS/FLI*; type 1 or 2 is indicated. Other E/F refers to other types of *EWS/FLI* fusion transcript (2). E/E, *EWS/ERG*. [b]As determined by conventional approaches; –, absence of metastasis. [c]Absence (–) or presence (+) of tumour cells.

evidenced by the analysis of either or both the primary and peripheral sites, peripheral tumour cells could be detected in 7/28 patients with the *EWS/FLI-1* transcript and in 2/4 with *EWS/ERG*. This suggests that the propensity of cells to circulate or to metastasise in bone marrow is not linked to the type of fusion transcript.

A total of 20 samples from 16 patients were studied at diagnosis (Table II). The presence of Ewing's cells in blood and bone marrow could be demonstrated in 4/13 and 2/5 cases respectively (Tables II and III). Two patients (cases nos. 16 and 17) with lung metastasis at diagnosis did not exhibit circulating Ewing's cells. In two patients (cases nos. 3 and 15), tumour cells could be detected in blood but not in the bone marrow. In these cases, circulating tumour cells might be unable to metastasise. Alternatively, localised metastatic sites in bone marrow may have escaped detection. In one patient (case no. 14) tumour cells were observed both in blood and in bone marrow. Interestingly, tumour cells could be detected in blood and/or bone marrow in 5/9 patients with a central primary. In contrast, no positive samples were observed in the seven patients with peripheral tumours (Table III).

After induction therapy, at the time of stem cell harvests for myelosuppressive therapy, 28 samples from 21 patients were analysed. None of these patients had evidence of metastasis at this time as detected by conventional techniques. Tumour cells could be detected in 1/8 bone marrow and in 2/18 stem cells harvests (Table II) but in neither of the two blood samples analysed (Table II). The three positive samples were collected from different patients.

Finally, two patients could be studied at relapse. Both had lung metastases. Tumour cells were detected in the bone marrow of one of them.

### Discussion

The existence of a tumour-specific genetic alteration (i.e. the *EWS/FLI-1* or *ERG* fusion transcripts) which can be specifically detected by PCR makes Ewing's tumour a model solid tumour for the detection of residual or minimal disease by RT-PCR. Such tumour-specific gene alterations resulting from chromosomal translocations are frequent in haematological malignancies and are widely used as markers for the detection of minimal disease. In solid tumours, tissue-specific markers have been used for the detection of tumour cells. Indeed, in neuroblastoma, tyrosine hydroxylase or PGP9.5 transcripts have been shown to be possible markers for the detection of tumour cells in blood or bone marrow from neuroblastoma patients (Naito *et al.*, 1991; Mattano *et al.*, 1992; Burchill *et al.*, 1994). In the same way, tyrosinase, keratin 19 and prostate-specific antigen have been used to detect tumour cells in patients with melanoma, breast carcinoma and prostate cancer respectively (Smith *et al.*, 1991; Moreno *et al.*, 1992; Datta *et al.*, 1994). Although the sensitivity of these assays is high, their specificity is reduced by the occurrence of false-positive results, which may be the result of illegitimate transcription in non-tumour cells.

We have developed a method which enables the detection of a small number of tumour cells in blood, bone marrow or peripheral stem cell harvests from patients with Ewing's tumour. In contrast to the tissue-specific transcripts previously used in solid tumours, this method relies on the detection of a specific tumour marker, thus ensuring an optimised specificity. Its sensitivity, assessed using reconstitution experiments, is over 1 cell per million mononuclear blood cells.

Detection of small numbers of cells by RT-PCR in biological specimens has to overcome two major drawbacks. The first is linked to the numerous steps necessary to process the sample from collection to final purification of RNAs. An error during any of these steps may result in degraded RNAs, giving rise to false-negative results. This emphasises the absolute need for an internal control to verify the quality of the RNA. In this series, this control was provided by amplification of the *EWS* transcript. This amplification was performed with one-tenth of the cDNAs used for detection of the fusion transcript with a single round of PCR. Only samples which promoted a strong amplification of *EWS* in these conditions were further analysed for tumour cell detection. The second major drawback is a result of the risk of cross-contamination linked to the use of PCR amplification techniques, especially when nested PCR strategies are performed. Such contamination may generate false-positive results. To avoid this problem, precautions included processing samples at different sites for RNA isolation, PCR amplification and electrophoresis of PCR products. Moreover, this risk was greatly reduced by processing the two rounds of PCR amplification without opening the tube. Finally, the observation that the type of fusion transcript is consistent between different tumour sites provides a good control for the specificity of the detection and lowers the risk of misinterpretation of the results as a result of sample cross-contamination given that the primary site and the peripheral site(s) were not processed at the same time.

This method was evaluated on biological samples from patients. At diagnosis, one-third of the patients presented with involvement of blood, bone marrow or both. None of these patients had detectable metastases as evaluated by chest CT scan and cytohistological examination of the bone marrow. This demonstrated that regional disease can be accompanied by circulating tumour cells or occult bone marrow metastasis. The impact of these findings on evolution of the disease will necessitate long-term follow-up of these and other patients.

As for other solid tumours, myeloablative therapy with stem cell grafting is now increasingly used for the treatment of Ewing's tumours, particularly in high-risk groups (Horowitz *et al.*, 1993). The recent demonstration that, in neuroblastoma, malignant cells present in the autologous bone marrow contribute to relapse (Hill *et al.*, 1994) suggests that an identical phenomenon could occur in Ewing's tumour and emphasises the need to test stem cell harvests for tumour cells before grafting. In Ewing's tumour, the method described here now enables evaluation of the prognostic implications of the presence of tumour cells in stem cell harvests.

It can be anticipated that a potentially important application of the method described here will be the regular follow-up of blood from patients treated for Ewing's tumour. A progressive increase in the number of circulating tumour cells might be indicative of a relapse. We are currently developing a competition assay which should enable precise determination of the number of tumour cells present in the sample.

Finally, the increasing number of tumour-specific genetic alterations described in solid tumours raises the possibility that the approach described here for the Ewing's family of tumours might be applied for other tumours characterised by specific fusion transcripts (Crozat *et al.*, 1993; Galili *et al.*, 1993; Rabbits *et al.*, 1993; Zucman *et al.*, 1993*b*; Clark *et al.*, 1994; Ladanyi and Gerald 1994).

**Acknowledgements**
We thank Marco Giovannini for critical review of the manuscript and the following clinicians for providing samples: Dr E Bouffet, Dr L Brugières, Dr M Brunat-Mentigny, Professor F Demeocq, Dr F Doz, Dr AM Gelot, Dr J Mercks, Professor J Lemerle, Dr H Pacquement, Dr E Quintana, Dr C Schmitt and Dr JL Stephan. This work was supported by grants from the Association pour la Recherche Contre le Cancer and the CNAMTS-INSERM.

### References

AURIAS A. RIMBAUT C. BUFFE D. DUBOUSSET J AND MAZABRAUD A. (1983). Chromosomal translocation in Ewing's sarcoma. *N. Engl. J. Med.*, **309**, 496-497.

BURCHILL SA. BRADBURY FM. SMITH B. LEWIS IJ AND SELBY P. (1994). Neuroblastoma cell detection by reverse transcriptase-polymerase chain reaction (RT-PCR) for tyrosine hydroxylase mRNA. *Int. J. Cancer*, **57**, 671-675.

CLARK J. ROCQUES PJ. CREW AJ. GILL S. SHIPLEY J. CHAN AM-L. GUSTERSON BA AND COOPER CS. (1994). Identification of novel genes, SYT and SSX, involved in the t(X;18)(p11.2;q11.2) translocation found in human synovial sarcoma. *Nature Genet.*, **7**, 502-508.

CLINE MJ. The molecular basis of leukemia. 1994. *N. Engl. J. Med.*, **330**, 328-336.

CROZAT AMAN AP. MANDAHL N AND RON D. (1993). Fusion of CHOP to a novel RNA-binding protein in human liposarcoma. *Nature*, **363**, 640-642.

DATTA YD. ADAMS PT. DROBYSKI WR. ETHIER SP. TERRY VH AND ROTH MS. (1994). Sensitive detection of occult breast cancer by the reverse-transcriptase polymerase chain reaction. *J. Clin. Oncol.*, **12**, 475-482.

DELATTRE O. ZUCMAN J. PLOUGASTEL B. DESMAZE C. MELOT T. PETER M. KOVAR H. JOUBERT I. DEJONG P. ROULEAU G. AURIAS A AND THOMAS G. (1992). Gene fusion with an ETS domain caused by chromosome translocation in human tumours. *Nature*, **359**, 162-165.

DELATTRE O. ZUCMAN J. MELOT T. SASTRE GARAU X. ZUCKER J-M. LENOIR GM. AMBROS PF. SHEER D. TURC-CAREL C. TRICHE TJ. AURIAS A AND THOMAS G. (1994). The Ewing family of tumours: a subgroup of small round cell tumours defined by specific chimeric transcripts. *N. Engl. J. Med.*, **331**, 294-299.

GALILI N. DAVIS RJ. FREDERICKS WJ. MUKHOPADHYAY S. RAUSCHER FJ. EMANUEL BS. ROVERA G AND BARR FG. (1993). Fusion of a fork head domain gene to PAX3 in the solid tumor alveolar rhabdomyosarcoma *Nature Genet*, **5**, 230-234.

GIOVANNINI M. BIEGEL JA. SERRA M. WANG J-Y. WANG J-J. NYCUM L. EMMANUEL BS AND EVANS GA. (1994). EWS-erg and EWS-FLI1 fusion transcript in Ewing's sarcoma and primitive neuroectodermal tumors with variant translocations. *J. Clin. Invest.*, **94**, 489-496.

HILL DR. SANTANA VM. ROBERTS WM. NILSON T. BOWMAN LC. KRANCE RA. HELSOP HE. MOEN RC. IHLE JN AND BRENNER KB. (1994). Direct demonstration that autologous bone marrow transplantation for solid tumors can return a multiplicity of tumorigenic cells. *Blood*, **84**, 380-383.

HOROWITZ ME. MALAWER MM. DELANEY TF AND TSOKOS MG. (1993). Ewing's sarcoma family of tumors: Ewing's sarcoma of bone and soft tissue and the peripheral primitive neuroectodermal tumors. In *Principles and Practice of Paediatric Oncology*. 2nd edn. Pizzo PA and Poplack DG. (eds) pp. 795-821. J.B. Lippincott: Philadelphia.

LADANYI M AND GERALD W. (1994). Fusion of EWS and WT1 genes in the desmoplastic small round cell tumor. *Cancer Res.*, **54**, 2837-2840.

MATTANO LA. MOSS TJ AND EMERSON SG. (1992). Sensitive detection of rare circulating neuroblastoma cells by the reverse transcriptase-polymerase chain reaction. *Cancer Res.*, **52**, 4701-4705.

MORENO JG. CROCE CC. FISHER R. MONNE M. VIHKO P. MULHOLLAND SG AND GOMELLA LG. (1992). Detection of hematogenous micrometastasis in patients with prostate cancer. *Cancer Res.*, **52**, 6110-6112.

NAITO H. KUZUMAKI N. UCHINO J-I. KOBAYASHI R. SHIKANO T. ISHIKAWA Y AND MATSUMOTO S. (1991). Detection of tyrosine hydroxylase mRNA and minimal neuroblastoma cells by the reverse transcription polymerase chain reaction. *Eur. J. Cancer*, **27**, 762-765.

RABBITS TH. FORSTER A. LARSON R AND NATHAN P. (1993). Fusion of the dominant negative transcription regulator CHOP with a novel gene FUS by translocation t(12;16) in malignant liposarcoma. *Nature Genet.*, **4**, 175-180.

SMITH B. SELBY P. SOUTHGATE J. PITTMAN K. BRADLEY C AND BLAIR GE. (1991). Detection of melanoma cells in peripheral blood by means of reverse transcriptase and polymerase chain reaction. *Lancet*, **338**, 1277-1229.

SORENSEN PHB. LESSNICK SL. LOPEZ-TERRADA D. LIU XF. TRICHE TJ AND DENNY CT. (1994). A second Ewing's sarcoma translocation, t(21;22) fuses the EWS gene to another ETS-family transcription factor, ERG. *Nature Genet.*, **6**, 146-151.

TURC-CAREL C. PHILIP I. BERGER MP. PHILIP T AND LENOIR GM. (1983). Chromosomal translocations in Ewing's sarcoma. *N. Engl. J. Med.*, **309**, 497-498.

ZUCMAN J. DELATTRE O. DESMAZE C. PLOUGASTEL B. JOUBERT I. MELOT T. PETER M. DEJONG P. ROULEAU G. AURIAS A AND THOMAS G. (1992). Cloning and characterisation of Ewing's sarcoma and peripheral neuroepithelioma t(11;22) translocation breakpoints. *Genes Chrom. Cancer*, **5**, 271-277.

ZUCMAN* J. MELOT T. DESMAZE C. GHYSDAEL J. PETER M. ZUCKER JM. TRICHE T. SHEER D. TURC-CAREL C. AMBROS P. COMBARET V. LENOIR G. AURIAS A. THOMAS G AND DELATTRE O. (1993*a*). Combinatorial generation of variable fusion proteins in peripheral primative neuroectodermal tumors. *EMBO J.*, **12**, 4481-4487.

ZUCMAN J. DELATTRE O. DESMAZE C. EPSTEIN AL. STENMAN G. FLETCHER C AND THOMAS G. (1993*b*). EWS and ATF-1 Gene fusion induced by the recurrent t(12;22) translocation in malignant melanoma of soft parts. *Nature Genet.*, **4**, 341-345.